# N-Acetyltransferase 10 Enhances Doxorubicin Resistance in Human Hepatocellular Carcinoma Cell Lines by Promoting the Epithelial-to-Mesenchymal Transition

**DOI:** 10.1155/2019/7561879

**Published:** 2019-07-01

**Authors:** Xiuming Zhang, Jiang Chen, Shi Jiang, Shilin He, Yanfeng Bai, Linghua Zhu, Rui Ma, Xiao Liang

**Affiliations:** ^1^Department of Pathology, The First Affiliated Hospital, College of Medicine, Zhejiang University, Hangzhou, Zhejiang 310003, China; ^2^Department of General Surgery, Sir Run Run Shaw Hospital, College of Medicine, Zhejiang University, Hangzhou, Zhejiang 310016, China; ^3^Department of Surgery, Zhejiang University Hospital, Zhejiang University, Hangzhou, Zhejiang 310027, China

## Abstract

**Background:**

N-Acetyltransferase 10 (NAT10) has been reported to be expressed at high levels in hepatocellular carcinoma (HCC); however, its role in chemoresistance is unclear. This study is aimed at investigating whether NAT10 regulates the epithelial-mesenchymal transition (EMT) and chemoresistance in HCC.

**Methods:**

HCC cell lines (Huh-7, Bel-7402, SNU387, and SNU449) were treated with remodelin, an inhibitor of NAT10, or transfected with small inhibitory RNAs (siRNAs) targeting NAT10 or Twist. The EMT was induced by hypoxia. The CCK-8 assay was used to quantify cell viability, the EdU incorporation assay to assess cell proliferation. siRNA knockdown efficiency and epithelial/mesenchymal marker expression were assessed by western blotting.

**Results:**

Knockdown of NAT10 using siRNA or inhibition of NAT10 using remodelin increased the sensitivity of HCC cell lines to doxorubicin; similar effects were observed in cells transfected with the Twist siRNA. Inhibition of NAT10 using remodelin also reversed the ability of doxorubicin to induce the EMT in HCC cells. Furthermore, inhibiting NAT10 reversed the hypoxia-induced EMT. Finally, we confirmed that combining doxorubicin with remodelin delayed tumor growth and reduced tumor cell proliferation in a mouse xenograft model of HCC.

**Conclusions:**

NAT10 may contribute to chemoresistance in HCC by regulating the EMT. The mechanism by which NAT10 regulates the EMT and doxorubicin sensitivity in HCC cells merits further investigation.

## 1. Introduction

Hepatocellular carcinoma (HCC) is the sixth most common malignant cancer worldwide. The 5-year overall survival rate for HCC is very low [1, 2], and the poor prognosis is mainly attributed to acquisition of chemoresistance during therapy [3]. However, the complicated cellular and molecular mechanisms that lead to chemoresistance in HCC remain unclear [4].

The epithelial-mesenchymal transition (EMT) is a complex, reversible progress resulting in the loss of epithelial cell adhesion and acquisition of a mesenchymal phenotype that plays a critical role in tissue regeneration, embryonic development, and inflammatory response [5–9]. During the EMT, epithelial markers such as E-cadherin are downregulated whereas mesenchymal markers such as vimentin and Twist are upregulated [10]. The EMT is implicated in the progression of cancer, and in recent decades, the EMT has been confirmed to play a role in the chemoresistance of various carcinomas, including HCC [11, 12]. The relationship between the EMT and drug resistance was first described by Mani et al., who inferred that blocking or reversing the EMT may cause chemoresistant cells to revert to chemosensitive cells [13].

We previously observed that N-acetyltransferase 10 (NAT10) is upregulated in HCC cell lines with a mesenchymal-like phenotype. Inhibition of NAT10 reduced cell migration and invasion ability and correlated with elevated E-cadherin expression and reduced vimentin expression. As E-cadherin and vimentin are canonical markers of the EMT, these data suggest that NAT10 may promote the EMT in HCC [14].

In the present study, we sought to clarify the role of NAT10 in the EMT and chemoresistance in HCC. We demonstrate that NAT10 plays a critical role in regulation of the EMT and chemoresistance in HCC; however, the underlying mechanisms require further investigation.

## 2. Material and Methods

### 2.1. Cell Culture

Huh-7 cells were cultured in Dulbecco's modified Eagle's media (DMEM) (Invitrogen, Carlsbad, CA, USA) supplemented with 10% fetal bovine serum (FBS) and 100 U/mL penicillin/streptomycin (Sigma, St. Louis, MO, USA). Bel-7402 cells were cultured in minimum essential medium (MEM) (Hyclone, Logan, UT, USA) supplemented with 10% FBS and 100 U/mL penicillin/streptomycin. SNU387 and SNU449 cells were cultured in Roswell Park Memorial Institute (RPMI-1640) medium (Gibco, Carlsbad, CA, USA) supplemented with 10% FBS and 100 U/mL penicillin/streptomycin. All cells were cultured at 37°C in a 5% CO_2_ incubator; 70–80% confluent cultures were used for all experiments. To induce hypoxia, HCC cells were exposed to hypoxic culture conditions (1% O_2_, 94% N_2_, and 5% CO_2_).

### 2.2. siRNA Transfection

The NAT10 siRNA (sc62660) and Twist siRNA (sc38604) were purchased from Santa Cruz Biotechnology Inc. (Santa Cruz Biotechnology, Dallas, TX, USA). The lyophilized oligonucleotides were reconstituted in RNase-free water to create 20 *μ*M stock solutions. Lipofectamine 2000 (Invitrogen) was used to transfect the siRNAs into Huh-7, Bel-7402, SNU387, and SNU449 cells according to the manufacturer's instructions. The transfected cells were incubated for 48 h before experiments.

### 2.3. Cell Counting Kit-8 (CCK-8) Assay

Huh-7, Bel-7402, SNU387, and SNU449 cells were seeded at 3000 cells per well in 96-well plates and incubated for 24 h, treated with and without doxorubicin/remodelin for 48 h, then 10 *μ*L of CCK-8 solution (Cell Counting Kit-8, Dojindo, Kumamoto, Japan) was added, and cells were incubated for 3 h. The OD (optical density) values were assessed using a MRX II microplate reader (Dynex, Chantilly, VA, USA).

### 2.4. Ethynyl Deoxyuridine (EdU) Assay

The Click-iT EdU Imaging Kit (Invitrogen) was used to assay cell proliferation following the manufacturer's protocol. Briefly, Huh-7, Bel-7402, SNU387, and SNU449 cells were incubated with doxorubicin at its IC_50_ with or without remodelin for 24 h, then 10 *μ*M EdU was added 2 h before fixation, permeabilization, and EdU staining. Nuclei were stained with 5 *μ*g/mL Hoechst 33342 (Invitrogen) for 30 min. Cell proliferation was assessed using the Click-iT EdU imaging kit (Invitrogen) according to the manufacturer's instructions.

### 2.5. Western Blotting

Cells were lysed with modified lysis buffer (50 mM Tris, 150 mM NaCl, 1% Triton X-100, and 0.5% deoxycholate). The BCA protein assay (Thermo Fisher Scientific, Rockford, IL, USA) was used to quantify protein concentrations. Samples containing approximately 40 *μ*g protein were separated by sodium dodecyl sulfate-polyacrylamide gel electrophoresis (SDS-PAGE; Bio-Rad, Hercules, CA, USA), transferred to PVDF membranes (Millipore, Billerica, MA, USA), then incubated with primary antibodies against NAT10 (1 : 1000, Santa Cruz), Twist (1 : 1000, Cell Signaling Technology, Danvers, MA, USA), vimentin (1 : 1000, Cell Signaling Technology, Danvers, MA, USA), E-cadherin (1 : 1000, Cell Signaling Technology), and GAPDH (1 : 1000, Cell Signaling Technology), followed by HRP-labeled secondary antibodies (1 : 2000, Cell Signaling Technology), then the bands were detected using chemiluminescent reagent (GE Healthcare, Piscataway, NJ, USA). The optical density of each band was quantified and expressed relative to the internal control GAPDH.

### 2.6. Immunofluorescent Staining

Huh-7, Bel-7402, SNU387, and SNU449 cells were seeded onto glass slides. At 48 h after transfection with the NAT10 siRNA or Twist siRNA or treatment with remodelin in the presence of doxorubicin or hypoxia, the cells were rinsed with PBS, fixed with 2% paraformaldehyde, permeabilized with 0.1% Triton X-100, blocked for 30 min in 10% BSA, and then incubated with an anti-E-cadherin monoclonal antibody (1 : 200; Cell Signaling Technology) or anti-vimentin monoclonal antibody (1 : 200; Cell Signaling Technology) overnight at 4°C. After three washes in PBS, the slides were incubated with goat anti-rabbit Cy3 as a secondary antibody (1 : 200; Cell Signaling Technology) for 1 h in the dark. After three further washes, the cells were stained with DAPI for 5 min to visualize nuclei and examined by confocal microscopy (Olympus, Tokyo, Japan).

### 2.7. Immunohistochemical Staining

Sections (4 *μ*m) from each block were deparaffinized in xylene and rehydrated in a descending alcohol series. Antigen retrieval was performed by heating in a pressure cooker in 10 mmol/L citrate buffer (pH 6.0). Endogenous peroxidase activity was blocked by incubation in 0.3% H_2_O_2_ for 15 min, followed by incubation with 5% serum to reduce nonspecific binding. Sections were incubated with an anti-vimentin monoclonal antibody (1 : 1000; Cell Signaling Technology), anti-E-cadherin monoclonal antibody (1 : 1000; Cell Signaling Technology), or anti-Ki-67 monoclonal antibody (1 : 500; Cell Signaling Technology) at 4°C overnight. After washing in phosphate-buffered saline (PBS), slides were incubated with horseradish peroxidase-conjugated rabbit-anti-mouse secondary antibody, developed using 3,3-diaminobenzidine (DAB) chromogen solution and counterstained with Mayer's hematoxylin. Negative controls were performed in parallel by replacing the primary antibody with nonspecific serum.

### 2.8. HuH-7 Mouse Xenograft Model

All animal experiments complied with the Guide for the Care and Use of the Animal Ethics Committee of Zhejiang University (Hangzhou, China). Male nude mice (3 to 4 weeks old, 16-20 g; Silaike Experimental Animal Centre; Shanghai, China) were housed under pathogen-free conditions and supplied with irradiated feed. Twenty-four mice were subcutaneously injected in the right axillary fossa with HuH-7 cells (1 × 10^6^) in 100 *μ*L PBS. Mice were randomly divided into four groups. Tumor length (*L*) and width (*W*) were measured every other day; tumor volumes were calculated using (*L* × *W*^2^)/2 [15]. Treatment was initiated when tumor volume reached 50-100 mm^3^. Mice received remodelin (3 mg/kg), doxorubicin (3 mg/kg), remodelin (3 mg/kg) combined with doxorubicin (3 mg/kg), or vehicle (control group; conformed to the National Institutes of Health Guide for Care and Use of Laboratory Animals (NIH Publications, No. 8023, revised 1978) equal volume of diluents) intraperitoneally every 2 days. After 2 weeks of treatment, mice were euthanized by cervical dislocation and tumors were dissected and weighed. Tumor proliferation was quantified using Ki-67 immunohistochemical staining.

### 2.9. Statistical Analysis

All data were analyzed using SPSS 18.0 (SPSS Inc., Chicago, IL, USA). Data are presented as mean ± SD. Two groups were compared using the Student *t*-test, and multiple groups were compared using one-way analysis of variance (ANOVA). Differences were considered significant at *P* < 0.05.

## 3. Results

### 3.1. Inhibition of NAT10 Enhances the Sensitivity of HCC Cell Lines to Doxorubicin

**Figure 1:**
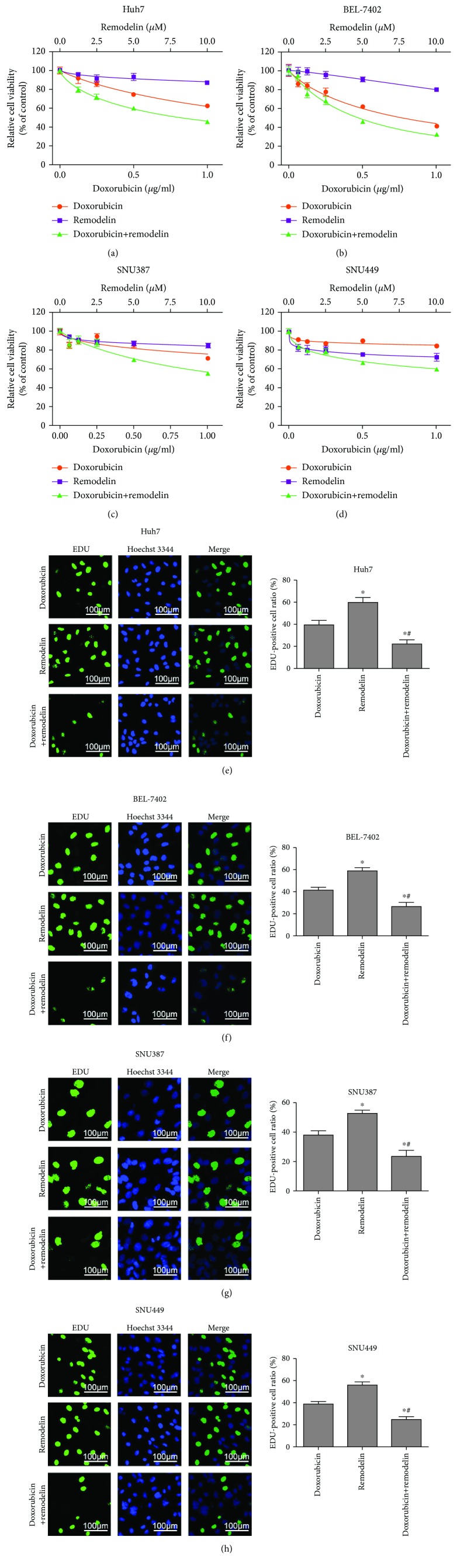
Inhibition of NAT10 using remodelin increases the chemosensitivity of HCC cell lines to doxorubicin. (a–d) CCK-8 assay of cell viability. (e–h) Representative images and quantification of EdU incorporation assay of cell growth and DNA synthesis. ^∗^*P* < 0.05, doxorubicin vs. control cells; ^#^*P* < 0.05, doxorubicin+remodelin vs. remodelin.

First, we examined the cell viabilities of HCC cells treated with doxorubicin and remodelin, an inhibitor of NAT10, for 48 h. The CCK-8 assay revealed that remodelin increased the doxorubicin sensitivity of all four cell lines (Figures 1(a)–1(d)). The EdU incorporation assay confirmed that the inhibition of NAT10 using remodelin decreased the proliferation of all four HCC cell lines when treated with doxorubicin (Figures 1(e)–1(h) and Table 1). These data indicate that NAT10 enhances the resistance of HCC cells to doxorubicin.

### 3.2. Knockdown of NAT10 Increases the Sensitivity of HCC Cell Lines to Doxorubicin

**Figure 2:**
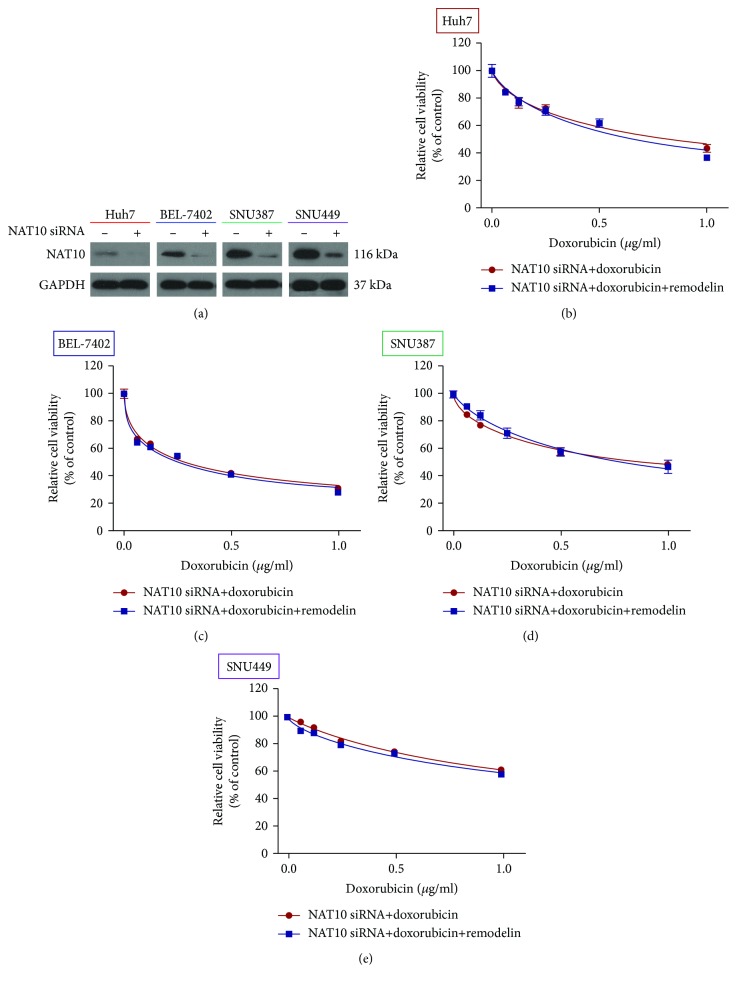
Knocking down and inhibiting NAT10 increase the chemosensitivity of HCC cell lines to doxorubicin. (a) Western blotting confirmation of NAT10 knockdown efficiency in siRNA-transfected cells. (b–e) CCK-8 assay of the viability of HCC cells treated with doxorubicin, the NAT10 siRNA, or doxorubicin and remodelin following knockdown of NAT10.

To further investigate the contribution of NAT10 to doxorubicin resistance in HCC, the four HCC cell lines were transfected with a NAT10 siRNA. Western blotting confirmed that NAT10 expression was almost completely knocked down in cells transfected with the NAT10 siRNA (Figure 2(a)). The CCK-8 assay revealed that the NAT10 siRNA had no significant effect on doxorubicin sensitivity compared to cells treated with the NAT10 inhibitor remodelin, confirming that NAT10 enhances the chemoresistance of HCC cells (Figures 2(b)–2(e)). Taken together, these data confirm that NAT10 enhances the resistance of HCC cells to doxorubicin.

### 3.3. NAT10 Promotes the EMT in HCC Cell Lines

**Figure 3:**
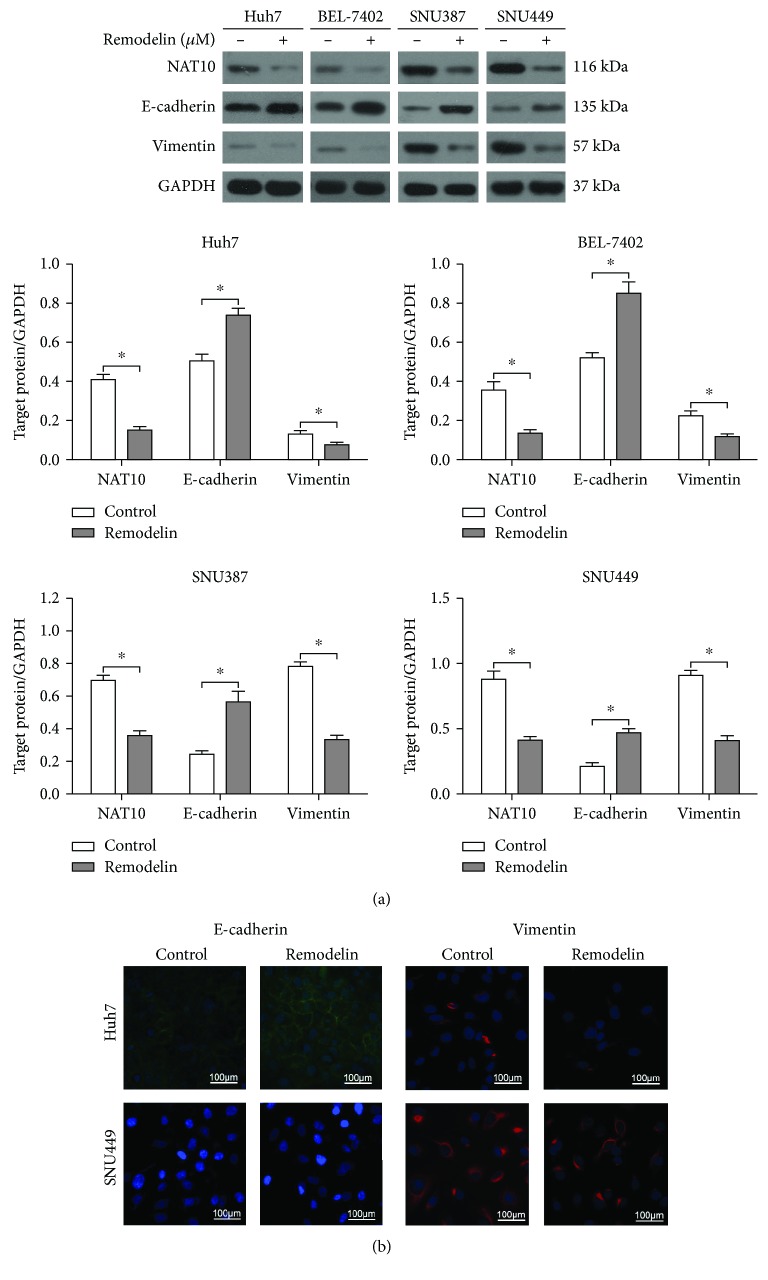
Inhibition of NAT10 using remodelin reverses the EMT in HCC cell lines. (a) Western blotting of NAT10, E-cadherin, and vimentin expression; ^∗^*P* < 0.05. (b) Immunofluorescence analysis of E-cadherin and vimentin expression in cells treated with or without remodelin (IC_50_ of remodelin in combination).

The expression of NAT10 and epithelial/mesenchymal markers were examined using western blotting to assess whether the inhibition of NAT10 using remodelin affects the EMT in HCC cells. Remodelin significantly increased E-cadherin expression and decreased NAT10 and vimentin expression in all four HCC cell lines (Figure 3(a)). The results of immunofluorescent staining were consistent with western blotting (Figure 3(b)), indicating that the inhibition of NAT10 using remodelin reversed the EMT phenotype in HCC cell lines.

### 3.4. Inhibition of NAT10 Using Remodelin Reverses the Doxorubicin-Induced EMT in HCC Cell Lines

**Figure 4:**
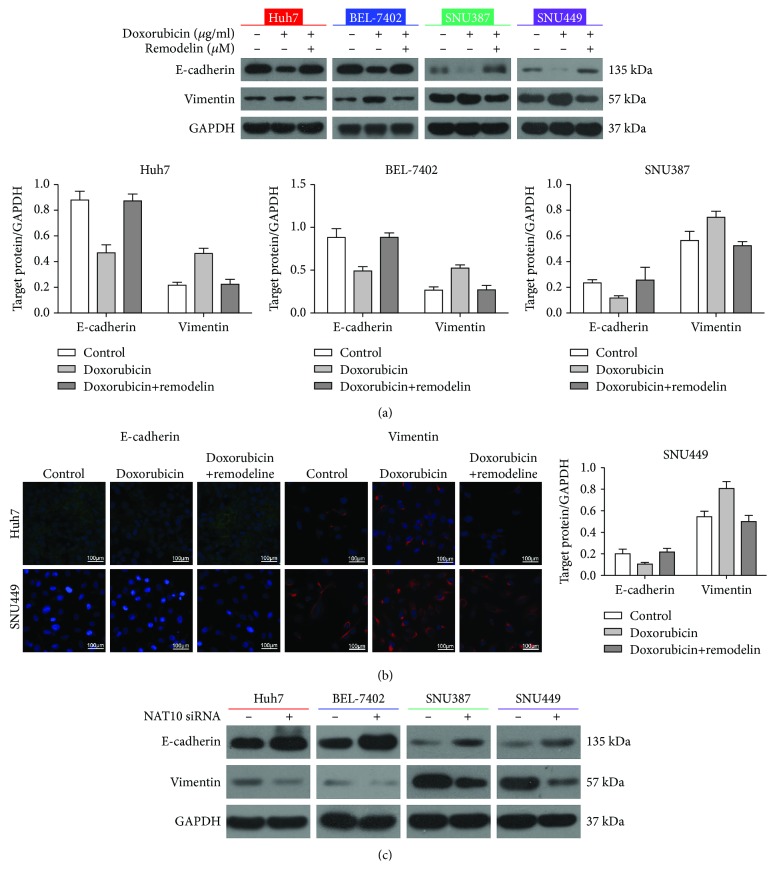
Inhibition of NAT10 reverses the doxorubicin-induced EMT in HCC cell lines. Western blotting and immunofluorescence analysis of the expression of EMT-related markers E-cadherin and vimentin in cells treated with doxorubicin or remodelin (a, b) or HCC cells transfected with the NAT10 siRNA (c).

Western blotting was performed to quantify the expression of EMT markers in HCC cell lines treated with doxorubicin in the presence and absence of the NAT10 inhibitor remodelin. Doxorubicin obviously reduced E-cadherin expression and increased vimentin expression, indicating that doxorubicin promotes the EMT in HCC cell lines. However, inhibition of NAT10 using remodelin reversed the ability of doxorubicin to promote the EMT, as indicated by the upregulation of E-cadherin and the downregulation of vimentin compared to control cells (Figure 4(a)). Immunofluorescent staining provided similar results as the western blot analysis (Figure 4(b)).

The knockdown efficiency of the NAT10 siRNA was confirmed by western blotting. Moreover, we observed that the NAT10 siRNA increased E-cadherin expression and reduced vimentin expression in the HCC cell lines (Figure 4(c)). Collectively, these results indicate that inhibition of NAT10 reverses the ability of doxorubicin to induce the EMT in HCC cells.

### 3.5. NAT10 Induces Doxorubicin Resistance by Promoting the EMT

**Figure 5:**
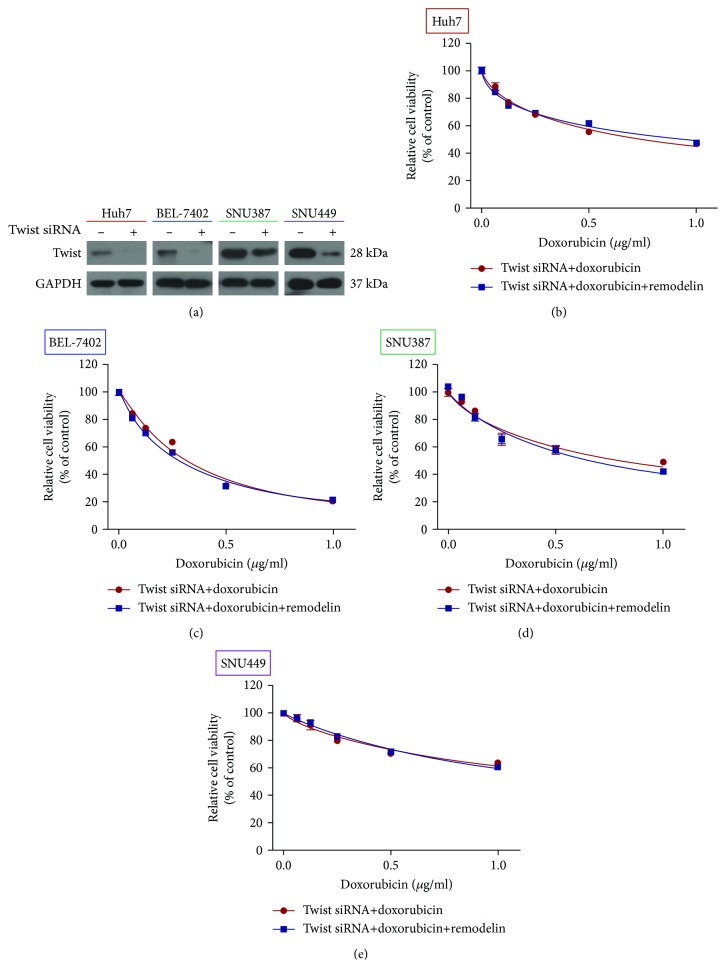
Effect of knocking down Twist using a siRNA on the chemosensitivity of HCC cell lines to doxorubicin. (a) Western blotting confirmation of Twist knockdown efficiency in siRNA-transfected cells. (b–e) CCK-8 assay of the viability of HCC cells treated with doxorubicin and remodelin following knockdown of Twist.

Twist functions as a critical transcription factor implicated in EMT and drug resistance [16]. We analyzed the effects of knocking down Twist on the sensitivity of HCC cells to doxorubicin. Western blotting confirmed that NAT10 expression was almost completely knocked down in cells transfected with the NAT10 siRNA (Figure 5(a)). The CCK-8 assay revealed the Twist siRNA had no significant effect on the sensitivity of HCC cells to doxorubicin compared to cells treated with remodelin (Figures 5(b)–5(e)), which confirmed that NAT10 induces doxorubicin resistance by promoting the EMT in HCC cell lines.

### 3.6. Inhibition of NAT10 Using Remodelin Reverses Hypoxia-Induced Doxorubicin Resistance and EMT in HCC Cell Lines

**Figure 6:**
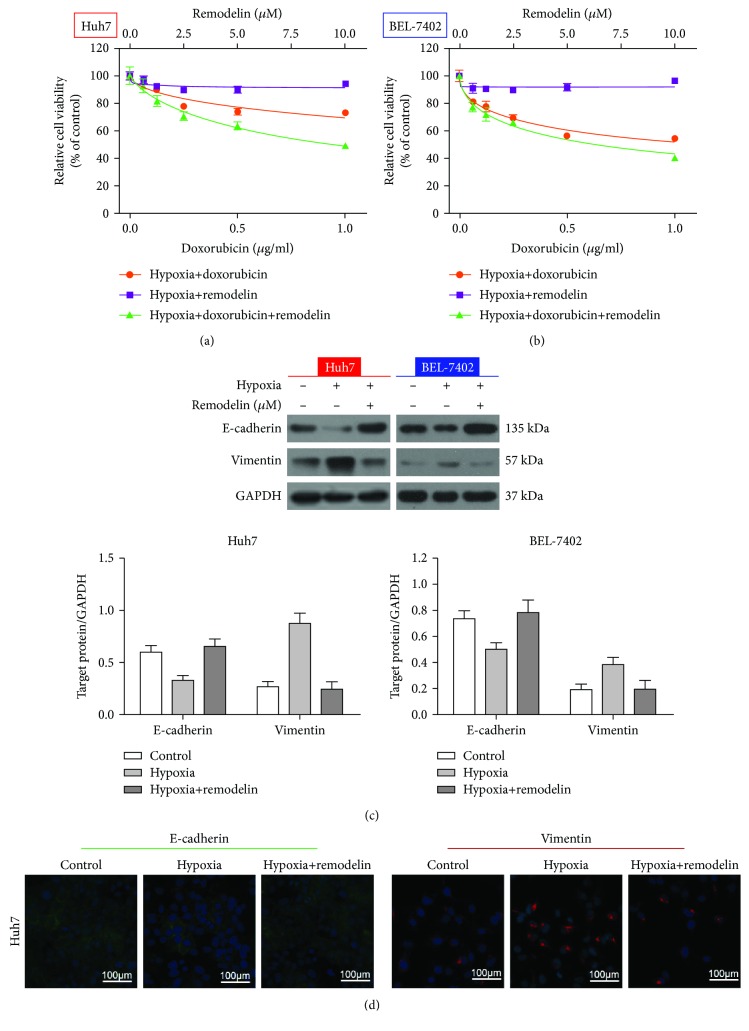
Inhibition of NAT10 reverses the hypoxia-induced EMT in HCC cell lines. (a, b) CCK-8 assay of Huh-7 and BEL-7402 cell viability treated with doxorubicin, remodelin, or doxorubicin plus remodelin under hypoxic conditions. (c) Western blotting analysis of E-cadherin and vimentin expression in Huh-7 and BEL-7402 cells subjected to hypoxic conditions. (c) Immunofluorescence analysis of E-cadherin and vimentin expression.

Hypoxia can induce the EMT in HCC cells [13]. Similarly, we observed that Huh-7 and BEL-7402 cells became more resistant to doxorubicin under hypoxic conditions. However, inhibition of NAT10 using remodelin attenuated hypoxia-induced doxorubicin resistance in HCC cells (Figures 6(a) and 6(b) and Table 2). Moreover, remodelin inhibited the hypoxia-induced downregulation of E-cadherin and upregulation of vimentin (Figure 6(c)). Immunofluorescent staining confirmed the western blot analysis. Taken together, this data indicates that NAT10 is required for the hypoxia-induced EMT and doxorubicin resistance in HCC cells.

### 3.7. Remodelin Enhances the Curative Efficacy of Doxorubicin in HCC *In Vivo*

**Figure 7:**
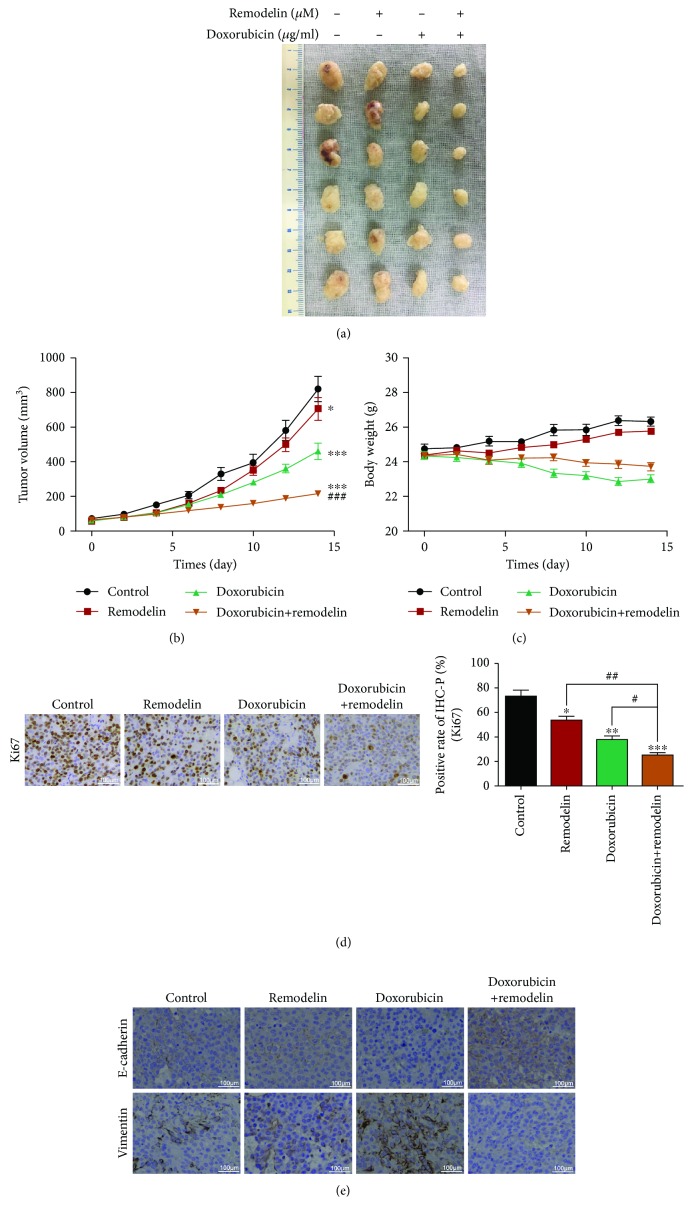
Remodelin enhances the efficacy of doxorubicin in a xenograft model of HCC in nude mice. (a) Mice were euthanized after 2 weeks of treatment and tumors were dissected. (b) Volume of tumor xenografts in the control group (black) and groups treated with remodelin (red), doxorubicin (green), or remodelin plus doxorubicin (brown). Relative tumor volume ratios are shown (% of original volume when therapy was initiated). (c) Body weight of mice in the control group (black) and groups treated with remodelin (red), doxorubicin (green), or remodelin plus doxorubicin (brown). Relative body weight ratios are shown (% of original volume when therapy was initiated). (d) Tumor proliferation rates. (e) Immunohistochemical analysis of expression of the EMT-related markers E-cadherin and vimentin in the xenograft tumors. Values are mean ± SD, *n* = 6; ^∗^*P* < 0.05, ^∗∗^*P* < 0.01, and ^∗∗∗^*P* < 0.001 for control vs. remodelin, doxorubicin, or remodelin plus doxorubicin; ^#^*P* < 0.05, ^##^*P* < 0.01, and ^###^*P* < 0.001 for doxorubicin plus remodelin vs. doxorubicin or remodelin alone.

To investigate the efficacy of combined doxorubicin and remodelin therapy in HCC *in vivo*, we subcutaneously injected HuH-7 cells into nude mice to establish a xenograft model of HCC; tumor growth was monitored in each treatment group every other day. Intraperitoneal injection of doxorubicin or remodelin alone for two weeks inhibited tumor growth. Interestingly, combined treatment with doxorubicin and remodelin led to more significant inhibition of tumor growth (Figures 7(a)–7(d)). The results showed that remodelin significantly inhibited tumor cell proliferation and thus enhanced the curative efficacy of doxorubicin in HCC *in vivo* (Figure 7(d)).

Immunohistochemical staining was performed to quantify the expression of EMT markers in the HCC xenograft tumors treated with doxorubicin, remodelin, or doxorubicin plus remodelin. Doxorubicin downregulated E-cadherin expression and upregulated vimentin expression, suggesting that doxorubicin promoted the EMT in the mouse model of HCC. However, remodelin attenuated the doxorubicin-induced EMT in tumor cells, as confirmed by upregulation of E-cadherin and downregulation of vimentin (Figure 7(e)).

## 4. Discussion

Deregulation of NAT10 has been reported in human cancer [14]. Our previous studies demonstrated that elevated NAT10 protein expression was associated with a poor prognosis in HCC [17]. Moreover, NAT10 is known to promote a more aggressive phenotype in HCC cells by inducing the EMT, as indicated by upregulation of mesenchymal markers such as E-cadherin and vimentin [14].

Chemotherapy is an effective postoperative therapy in a variety of cancers, and doxorubicin is widely used as a first-line chemotherapy agent for HCC [4]. However, acquisition of drug resistance to doxorubicin is a major factor that leads to recurrence in HCC [18]. In the present study, we investigated whether NAT10 is involved in doxorubicin resistance in HCC. Here, we report that inhibition of NAT10 using remodelin or a NAT10 siRNA increased the sensitivity of HCC cell lines to doxorubicin.

The EMT is well-recognized as an important factor associated with drug resistance in cancer [19]. We found that inhibition of NAT10 using remodelin inhibited the EMT and downregulated the expression of NAT10, E-cadherin, and vimentin in all four HCC cell lines. E-cadherin and vimentin are well-recognized markers of the mesenchymal phenotype and play key roles in the EMT by maintaining the intercellular junctions of epithelial cancer cells [20, 21]. Additionally, inhibition of NAT10 using remodelin reversed the doxorubicin-induced EMT in HCC cells. In agreement with these observations, knockdown of Twist, a transcriptional repressor of E-cadherin [22], also prevented the EMT, as indicated by upregulation of E-cadherin and downregulation of vimentin. Overall, these results indicate that NAT10 confers doxorubicin resistance in HCC by promoting the EMT.

Moreover, we observed that hypoxia could induce the EMT in HCC cells, and Huh-7 and BEL-7402 cells became more resistant to doxorubicin under hypoxic conditions. Another study demonstrated that curcumin inhibits the hypoxia inducible factor-1*α*-induced EMT in HCC cells [23]. Interestingly, the inhibition of NAT10 using remodelin restored doxorubicin sensitivity to HCC cells exposed to hypoxic conditions. Moreover, hypoxia-induced downregulation of E-cadherin and upregulation of vimentin could be reversed by inhibition of NAT10 in Huh-7 and BEL-7402 cells. The *in vivo* xenograft models confirmed that remodelin significantly inhibited tumor proliferation and enhanced the curative efficacy of doxorubicin in HCC. Collectively, these data indicate that inhibition of NAT10 using the siRNA or remodelin increases doxorubicin sensitivity and prevents the EMT in HCC cells.

## 5. Conclusions

This study demonstrates that NAT10 plays important roles in the regulation of the EMT and doxorubicin sensitivity in HCC cells. These observations indicate that NAT10 represents a potential target for overcoming chemoresistance in HCC and provides a rationale for combining remodelin with doxorubicin in the treatment of HCC. The mechanism by which NAT10 regulates the EMT and doxorubicin sensitivity in HCC cells merits further investigation.

## Figures and Tables

**Table 1:** IC_50_ values and statistical analyses of doxorubicin (DOX) and remodelin (Remo) treatments in HCC cell lines.

	IC_50_^a^			
	Dox	Remo	Dox+Remo	Combination index
Huh7	1.687 (0.9574-2.416)	465.3 (-1676-2607)	Dox 0.8271 (0.6688-0.9854) Remo 8.271 (6.688-9.854)	0.5080

BEL-7402	0.7633 (0.5844-0.9422)	29.33 (-1.089-59.76)	Dox 0.4677 (0.3895-0.5459) Remo 4.677 (3.895-5.459)	0.7721

SNU-387	9.178 (-6.944 to 25.30)	1752 (-4078-7583)	Dox 1.313 (0.8707-1.756) Remo 13.13 (8.707-17.56)	0.1505

SNU-449	23969 (-249863-297802)	1036 (-4182-6255)	Dox 2.379 (1.219-3.539) Remo 23.79 (12.19-35.39)	0.0230

^a^IC_50_ values show doxorubicin (*μ*g/mL) and remodelin concentration (*μ*M) (concentration, mean (95% confidence intervals)).

**Table 2:** IC_50_ values and statistical analyses of doxorubicin (DOX) and remodelin (Remo) treatments in HCC cell lines under hypoxia condition.

	IC_50_^a^			
	DOX	Remo	Dox+Remo	Combination index
Huh7	4.327 (0.6257-8.029)	Value too large	Dox 0.9489 (0.6332-1.265) Remo 9.489 (6.332-12.65)	0.22

BEL-7402	1.171 (0.7159-1.626)	Value too large	Dox 0.6343 (0.4754-0.7933) Remo 6.343 (4.754-7.933)	0.54

^a^IC_50_ values show doxorubicin (*μ*g/mL) and remodelin concentration (*μ*M) (concentration, mean (95% confidence intervals)).

## Data Availability

Data sharing is not applicable for this article as no datasets were generated or analyzed during the current study.

## Abbreviations

HCC: Hepatocellular carcinoma
EMT: Epithelial-mesenchymal transition
NAT10: N-Acetyltransferase 10
CCK-8: Cell counting kit-8
ANOVA: One-way analysis of variance
IF: Immunofluorescent staining
IHC: Immunohistochemical staining.
